# Supplementary material for: Moving to 3D: relationships between coral planar area, surface area and volume
Source: PeerJ. 2018 Feb 6;6:e4280. doi: 10.7717/peerj.4280 (PMC5806594; doi:10.7717/peerj.4280)
Supplement: Supplemental Information 1 [file peerj-06-4280-s001.docx]

**Table S1: Glossary of abbreviations**

| **Abbreviation** | **Definition** |
| --- | --- |
| TSA | Total surface area |
| LSA | Surface area consisting of corallites |
| CT scan | Computed tomography scan |
| PL TSA | Total surface area of specimen based on planar photography |
| PL LSA | Surface area of the specimen consisting of corallites, according to planar photography |
| PH TSA | Total surface area of specimen based on photogrammetry |
| PH LSA | Surface area of specimen consisting of corallites, according to photogrammetry |
| PH Vol | Volume of the specimen according to photogrammetry |
| CT TSA | Total surface area of specimen based on computed tomography scans |
| CT LSA | Surface area of specimen consisting of corallites, according to computed tomography scans |
| CT Vol | Volume of the specimen according to computed tomography scans |

**Table S2:** ID number, species, morphotype, number of photographs used for photogrammetry and dimensions for each coral skeleton used. The images of each coral are from photogrammetry or photographs used for measuring planar area. The images were selected to provide the best view of characteristics that are useful for species identification.

| ID number | Species | Morphotype | Alternative Morphotype (according to AIMS, 2013) | Mean number of images used for photogrammetry | Bounding box  (LxWxH,  in cm) | Image |
| --- | --- | --- | --- | --- | --- | --- |
| 1 | Acropora nasuta | Branching | Corymbose | 128 | 19.1 x 20.8 x 15.4 | 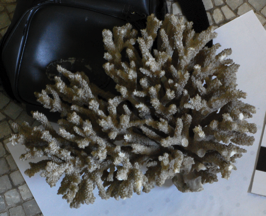 |
| 2 | Acropora polystoma | Branching | Corymbose | 113 | 21.7 x 27.0 x 17.0 | 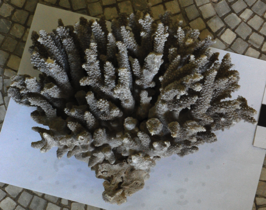 |
| 3 | Acropora hyacinthus | Branching | Table | 132 | 22.0 x 18.4 x 17.0 | 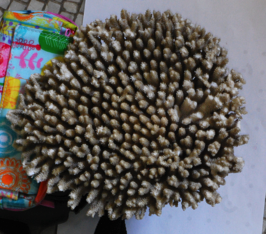 |
| 4 | Symphyllia radians | Massive | Hemispherical | 48 | 9.4 x 11.9 x 6.1 | 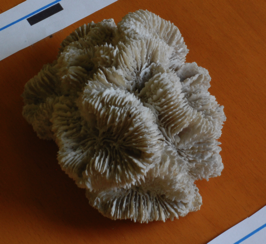 |
| 5 | Porites spp | Massive | Stumpy | 112 | 17.2 x 17.3 x 8.8 | 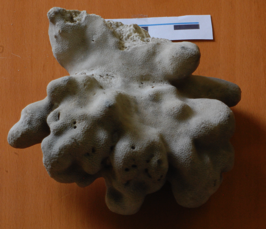 |
| 6 | Montipora spp. | Massive | Stumpy | 77 | 16.3 x 18.1 x 8.1 | 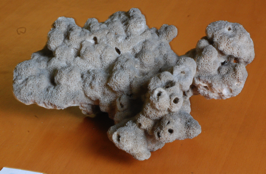 |
| 7 | Favia stelligera | Massive | Spherical | 82 | 11.7 x 14.1 x 6.2 | 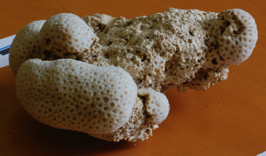 |
| 8 | Goniastrea retiformis | Massive | Hemispherical | 140 | 20.9 x 22.1 x 12.8 | 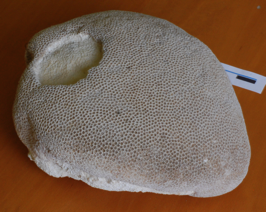 |
| 9 | Porites spp | Massive | Stumpy | 62 | 11.0 x 11.2 x 8.8 | 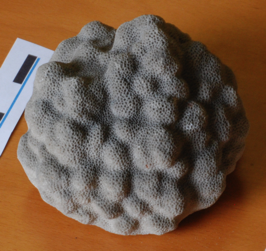 |
| 10 | Podabacia crustacea | Encrusting | Plate | 97 | 22.5 x 19.1 x 5.8 | 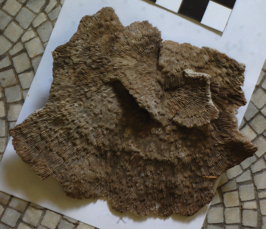 |
| 11 | Echinopora gemmacea | Encrusting | Laminar | 78 | 12.5 x 17.0 x 4.3 | 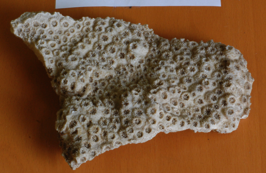 |
| 12 | Acropora hyacinthus | Branching | Table | 94 | 22.3 x 19.2 x 4.3 | 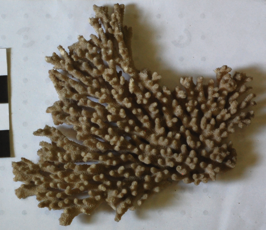 |
| 13 | Leptoria irregularis | Encrusting | Submassive | 94 | 25.1 x 21.8 x 12.9 | 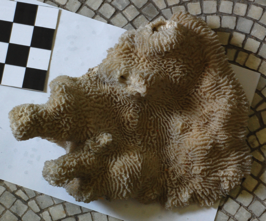 |
| 14 | Platygyra crosslandi | Encrusting | Plate | 74 | 20.4 x 11.9 x 11.1 | 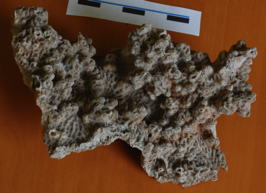 |
| 15 | Acropora formosa | Branching | Arborescent | 93 | 22.1 x 18.5 x 9.6 | 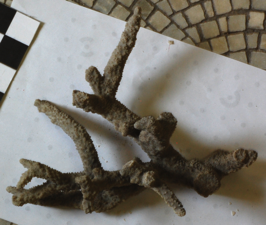 |
| 16 | Symphyllia recta | Massive | Hemispherical | 67 | 6.7 x 6.8 x 3.9 | 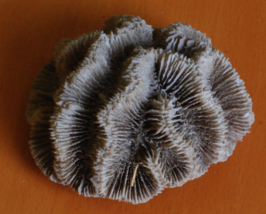 |
| 17 | Porites spp | Massive | Stumpy | 82 | 6.5 x 7.5 x 6.4 | 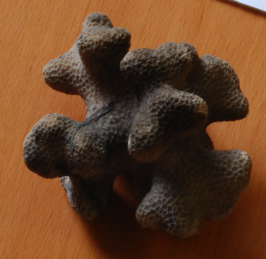 |
| 18 | Acropora hyacinthus | Branching | Table | 68 | 16.3 x 14.9 x 4.9 | 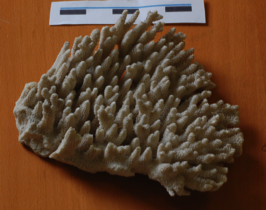 |
| 19 | Acropora cytherea | Branching | Table | 105 | 11.1 x 8.3 x 4.6 | 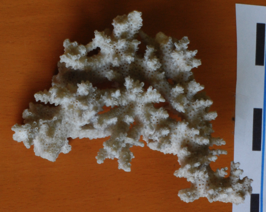 |
| 20 | Goniastrea retiformis | Massive | Hemispherical | 50 | 13.1 x 7.3 x 9.7 | 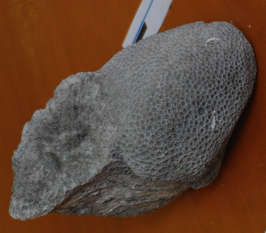 |
| 21 | Acropora formosa | Branching | Arborescent | 58 | 6.9 x 9.5 x 16.6 | 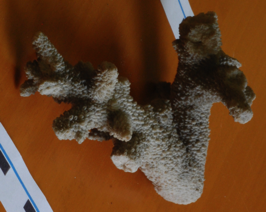 |
| 22 | Acropora carduus | Branching | Bottlebrush | 97 | 6.0 x 7.7 x 10.5 | 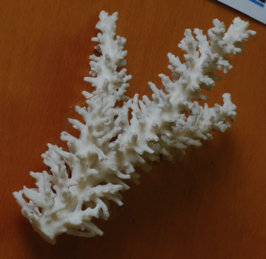 |

| Specimen 1  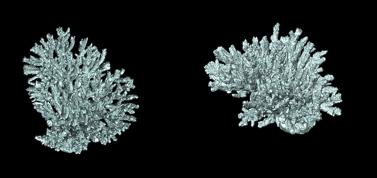  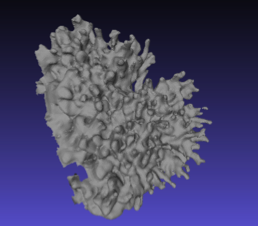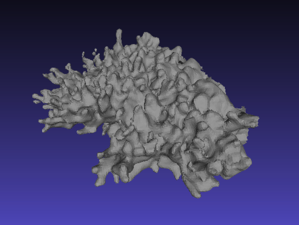 | Specimen 3  **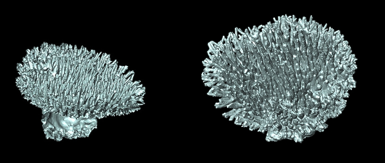**  **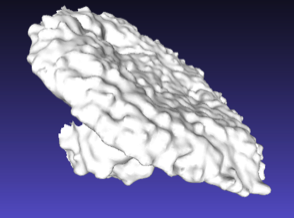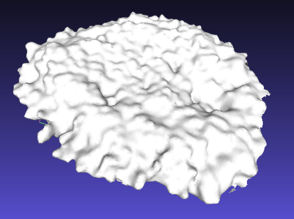** |
| --- | --- |
| Specimen 4  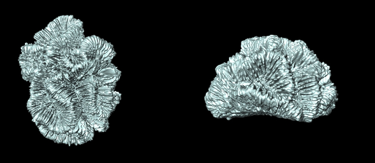  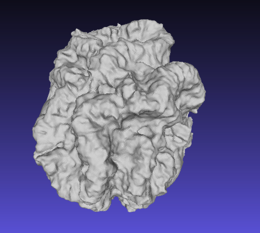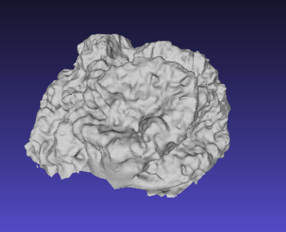 | Specimen 8  **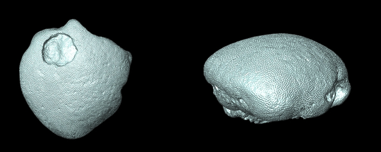**  **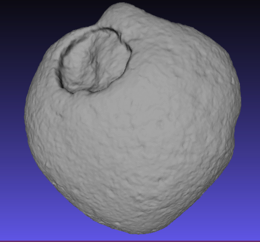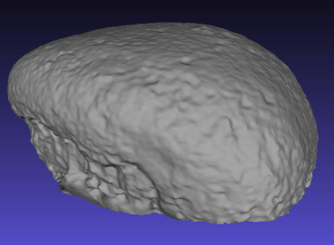** |
| Specimen 11  **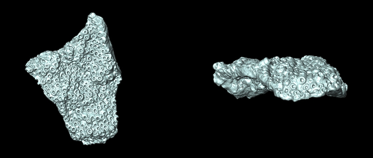**  **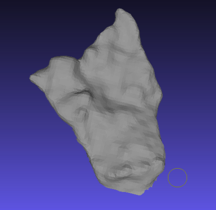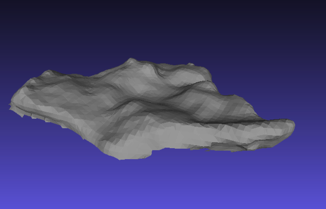** | Specimen 15  **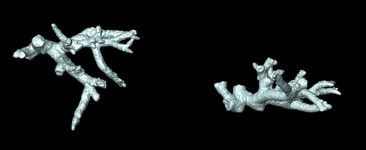**  **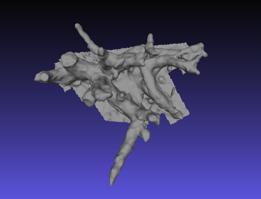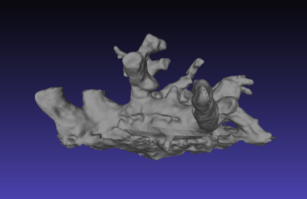** |
| Specimen 17  **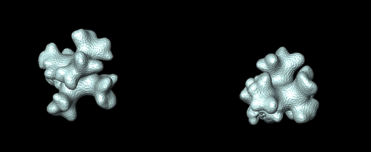**  **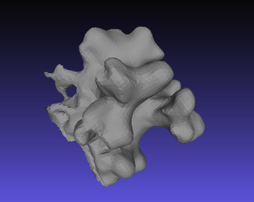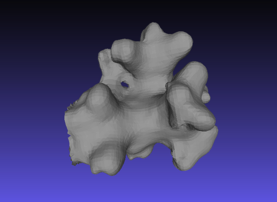** | Figure S1: 3D reconstructions of selected coral specimens at similar orientations respectively based on X-ray Computed Tomography (black background) and Photogrammetry (blue background). |
